# Flavonoids improve neurotransmitters for Parkinson’s treatment: mechanism and therapeutic potential

**DOI:** 10.3389/fphar.2026.1799142

**Published:** 2026-04-09

**Authors:** Yue Zhang, Yifan Bu, Mingrong Song, Zhihua Hao, Jing Chen

**Affiliations:** 1 The First Affiliated Hospital of Heilongjiang University of Chinese Medicine, Heilongjiang University of Chinese Medicine, Harbin, China; 2 College of Basic Medical and Sciences, Heilongjiang University of Chinese Medicine, Harbin, China

**Keywords:** bioactivity, extraction techniques, flavonoid, neurotransmitter, Parkinson

## Abstract

Parkinson’s disease (PD) is recognized as the second most common neurodegenerative disorder worldwide, and it stands out as one of the neurological conditions exhibiting the fastest rise in prevalence, disability, and economic consequences. While the degeneration of dopaminergic neurons and the depletion of striatal dopamine are fundamental to the classic motor symptoms of PD, growing evidence suggests that PD is a multifaceted multisystem disorder marked by extensive impairment across various neurotransmitter systems. Beyond dopaminergic impairment, serotonergic, glutamatergic, γ-aminobutyric acid (GABA)ergic and cholinergic pathways are profoundly disrupted during disease progression, contributing to motor and non-motor symptoms that respond poorly to dopamine-centred therapies. These limitations underscore an unmet need for multi-target therapeutic strategies capable of restoring broader neurotransmitter homeostasis. Flavonoids represent a varied group of polyphenolic compounds sourced from plants and have been recognized as potential neuropharmacological candidates due to their multifaceted biological activities and advantageous safety characteristics. Flavonoids not only possess antioxidant and anti-inflammatory characteristics, but they also influence intracellular signaling pathways, mitochondrial activity, neuroinflammation, and synaptic plasticity. Additionally, many of these compounds have the ability to traverse the blood-brain barrier. A growing body of preclinical evidence suggests that representative flavonoids—including baicalein, quercetin, apigenin, luteolin and EGCG—exert neuroprotective effects in experimental PD models by preserving dopaminergic neurons, attenuating α-synuclein pathology, modulating monoamine metabolism and suppressing glutamate-driven excitability.In this Review, we synthesize current evidence that flavonoids can ameliorate neurotransmitter dysfunction in PD. Focusing on dopamine, serotonin, glutamate and acetylcholine, we integrate experimental findings to highlight the multi-target regulatory capacity of flavonoids. We also discuss key pharmacokinetic limitations, nanodelivery strategies, toxicological considerations and translational challenges.

## 1 Introduction

Parkinson’s disease (PD) is known as the second-most prevalent neurodegenerative condition worldwide, primarily marked by issues related to movement. The main pathological characteristics involve the progressive loss of dopaminergic neurons in the substantia nigra area of the midbrain, along with the accumulation of α-synuclein within Lewy bodies found in neurons (MacLeod et al., 2013; de Lau and Breteler, 2006). However, this classical definition is increasingly inadequate to capture the clinical complexity of PD. PD, recognized as the most rapidly increasing neurological condition, is anticipated by certain estimates to influence around 12 million people globally by the year 2040 (Chen, 2010); This trajectory will impose a substantial burden on patients, families and society at large (Global, 2018). Age is the strongest risk factor for PD, and sex also influences its epidemiology, with a higher prevalence in men than in women (Wright Willis et al., 2010; Van Den Eeden et al., 2003). On one side, highly penetrant mutations in genes such as SNCA (alpha-synuclein), LRRK2 (leucine-rich repeat kinase 2), PRKN/PARK2 (parkin), PINK1 (PTEN-induced kinase 1), and GBA (glucocerebrosidase) can result in familial and/or early-onset forms of PD. On the other hand, numerous common variants distributed across at least ∼90 loci contribute to overall risk for sporadic PD through a cumulative polygenic effect. Notably, currently identified genetic determinants still account for only a fraction of the estimated heritability, and the remaining “missing heritability” is likely to be uncovered through larger studies and more diverse, multi-ancestry cohorts. Genetic discovery is increasingly enabling genotype-informed risk prediction, aetiology-based stratification and target-directed interventions, thereby laying a foundation for precision medicine in PD (Bandres-Ciga et al., 2020). Over decades of investigation, our understanding of PD has undergone a profound shift—from a framework centred largely on a pure motor disorder to one that recognizes PD as a complex multisystem disease, with marked heterogeneity and complexity across its clinical manifestations, pathophysiology and genetic architecture (Bloem et al., 2021). Current concepts of PD pathogenesis encompass mitochondrial dysfunction and lysosomal impairment, as well as more recent emphasis on gut-derived adaptive immune dysregulation and chronic inflammatory responses—processes that have each been shown to accelerate pathological progression (Morris et al., 2024). The initial description of these symptoms was made in 1817 by Dr. James Parkinson, who referred to it as “shaking palsy” (Parkinson, 2002). Later studies have indicated that PD often presents with various neuropsychiatric symptoms, such as apathy and depression (Han et al., 2018). Moreover, most patients develop at least one form of autonomic dysfunction, affecting systems such as cardiovascular regulation, urinary control and gastrointestinal function (Han et al., 2022). An imbalance of neurotransmitters significantly influences the pathophysiology of PD. In addition to dopamine, there are notable disruptions in various neurotransmitter systems, such as acetylcholine, serotonin (5-hydroxytryptamine; 5-HT), and noradrenaline, within the PD-affected brain. Dopamine-independent dysfunction in these circuits can exacerbate both motor and non-motor symptoms, resulting in a substantial decline in quality of life (Marino et al., 2025; Vaswani et al., 2025). PD is now widely viewed not as a disorder driven by a single neurotransmitter deficit, but as a complex neurofunctional syndrome arising from disrupted synaptic interplay among multiple transmitter systems (for example, dopamine and 5-HT) within key circuits such as the striatum and prefrontal cortex (Marino et al., 2025).

Given the hallmark dopaminergic deficit in PD, levodopa (L-DOPA)–based dopamine replacement has remained the standard therapy for motor symptoms since the 1960s (MacLeod et al., 2013; Fahn, 2008). Levodopa (L-DOPA), acting as a dopamine (DA) precursor, has the ability to traverse the blood-brain barrier. This results in intermittent elevations of striatal dopamine levels in a manner that is not physiological, leading to significant enhancements in motor function, especially during the early stages of the disease. However, while these treatments can be beneficial in the short term, extended use may lead to negative side effects, including motor issues such as dyskinesia (Bastide et al., 2015; Olanow and Schapira, 2013). Addressing motor fluctuations and dyskinesia induced by levodopa (LID) has emerged as a significant focus in the management of PD. In addition, dopamine receptor agonists are associated with adverse effects—including withdrawal syndromes, excessive daytime sleepiness and impulse-control disorders—which substantially constrain their clinical use (Chaudhuri et al., 2015). In recent years, studies of natural products such as quercetin, curcumin and EGCG in psychiatric disorders have suggested that these agents can exert multi-target, network-level effects through convergent mechanisms, including modulation of dopaminergic or glutamatergic signalling, activation of the BDNF pathway, and suppression of neuroinflammation and oxidative stress. This conceptual framework offers a fresh perspective: instead of relying solely on dopamine replacement, natural compounds may enable coordinated modulation of neurotransmitter dysregulation, mitochondrial impairment, and inflammation, which could be better suited to slowing disease progression and reducing the overall symptom burden (Ji et al., 2025).

Polyphenols are naturally occurring phytochemicals; collectively, they comprise more than 8,000 compounds and are broadly classified into flavonoids and non-flavonoid polyphenols (Ulusoy and Sanlier, 2020). Flavonoids represent a significant class of naturally occurring substances characterized by varied phenolic structures, commonly found in a range of fruits, vegetables, grains, and other foods derived from plants. In terms of structure, flavonoids are defined by a backbone consisting of C6–C3–C6, which includes three aromatic rings along with numerous hydroxyl groups. They can be classified into various categories such as isoflavonoids, neoflavonoids, and other subclasses like flavones, flavonols, flavanones, flavan-3-ols (including catechins), anthocyanins, and chalcones. These natural compounds are appreciated for their beneficial health effects, and flavonoid substances have become essential elements in nutraceuticals, pharmaceutical formulations, and medicine. The importance of this is mainly attributed to their capacity to interact with various biological targets and affect the roles of essential cellular enzymes (Panche et al., 2016; Wang et al., 2022). Flavonoid compounds have attracted considerable attention from both the scientific community and the public owing to their potential benefits for human health, including reported roles in regulating platelet aggregation, limiting lipid peroxidation and promoting mitochondrial biogenesis (Deepika and Maurya, 2022). A significant amount of research indicates that diet abundant in flavonoids not only provide neuroprotection but also help mitigate molecular and cellular changes that arise, as various neurodegenerative diseases progress. Additionally, many flavonoids can traverse the blood-brain barrier (Szulc et al., 2024). The actions of neuromodulation involve several neurotransmitter systems, such as noradrenergic, serotonergic (5-HT), GABAergic, and dopaminergic pathways (Pannu et al., 2021). Current evidence indicates that flavonoids share a common C6–C3–C6 scaffold yet exhibit substantial structural diversity, occurring widely in plant-based foods, often as glycosides or conjugates with other constituents. Their physicochemical properties and biological activities are strongly shaped by structural features such as hydroxylation patterns and glycosylation. Following oral administration, flavonoid absorption in the gastrointestinal tract is generally limited and depends on enzymatic processing in the small intestine and hydrolysis by the gut microbiota, followed by extensive biotransformation during enterohepatic circulation—including glucuronidation, sulfation and methylation. Consequently, flavonoids circulate predominantly as a spectrum of metabolites that mediate much of their biological activity, and overall bioavailability tends to be low. Notably, flavonoids and the gut microbiota engage in bidirectional crosstalk: microbial communities contribute to flavonoid degradation and conversion, whereas flavonoids and their metabolites can, in turn, remodel microbial composition and function. Given their intrinsic instability and bioavailability constraints, advanced delivery platforms—such as nanoformulations—are being developed to enhance solubility, stability and tissue targeting, thereby improving the prospects for harnessing flavonoids in disease prevention and therapy (Chen et al., 2022; Steed et al., 2017; Chalet et al., 2018). Taken together, flavonoid compounds offer a conceptual shift for PD therapeutics. In this review, we adopt a multi-neurotransmitter perspective—spanning dopaminergic, serotonergic (5-HT), glutamatergic, GABAergic and cholinergic systems—to systematically synthesize experimental and clinical evidence that flavonoids improve neurotransmitter function and modulate both motor and non-motor symptoms in PD, and to consider the potential advantages conferred by their multi-target, integrated regulatory actions.

## 2 Flavonoids

### 2.1 Baicalein

Baicalein (5,6,7-trihydroxyflavone) is a yellow, crystalline flavonoid (Rossi et al., 2001). It is a naturally occurring flavone widely distributed across plants, with Scutellaria baicalensis (Huangqin) as its principal source; baicalein has also been detected in the seeds and fruits, as well as the roots and leaves, of Zanthoxylum (Chinese prickly ash). As one of the major bioactive constituents of S. baicalensis, baicalein exhibits a broad spectrum of pharmacological activities and has therefore attracted considerable research interest. The medicinal use of S. baicalensis can be traced back to China’s Han dynasty, when it was used to treat diverse conditions, including inflammation, infection and gastrointestinal disorders (Wang et al., 2018). Studies have shown that baicalein, a natural flavonoid compound, possesses anti-inflammatory, immunomodulatory, and cytoprotective pharmacological activities, and has attracted considerable attention in multiple research fields, including cardiovascular diseases, liver diseases, and neurodegenerative disorders (Hu et al., 2022). Baicalein has also been reported to have anxiolytic and mild sedative effects (Gasiorowski et al., 2011). Evidence further indicates that baicalein can modulate neurotransmitter systems, including glutamatergic signalling, and may influence 6-hydroxydopamine (6-OHDA)-related neurotoxicity (Yu et al., 2012).

Although baicalein exhibits substantial bioactivity and therapeutic promise across diverse pharmacological studies, its clinical translation remains markedly constrained, largely owing to its extremely low aqueous solubility and poor oral absorption (Pi et al., 2019). Baicalein is found in two polymorphic variations, α and β; the β variant demonstrates significantly greater bioavailability compared to the α variant and has the ability to enhance the solubility of baicalein (Tian et al., 2012; Song et al., 2021). With respect to *in vivo* absorption, baicalein shows limited uptake in the colon, whereas absorption is comparatively more favourable in the stomach and small intestine (Taiming and Xuehua, 2006). Baicalein undergoes extensive glucuronidation during intestinal absorption, and this gut-associated first-pass metabolism likely contributes substantially to its low oral bioavailability (Zhang et al., 2005). Moreover, the *in vivo* delivery and pharmacological performance of baicalein are jointly constrained by its low aqueous solubility, pH-dependent behaviour and temperature sensitivity (Li et al., 2018; Bhatia et al., 2022).

**FIGURE 1:**
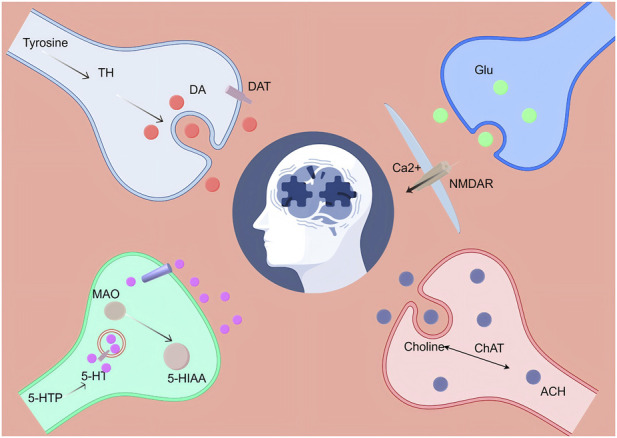
Chemical formula.

To improve baicalein bioavailability, extensive efforts have been undertaken. For example, researchers have used high-pressure homogenization to prepare baicalein–nicotinamide (BE–NCT) nanococrystals, which effectively enhance baicalein dissolution and oral bioavailability (Pi et al., 2019). Solutol HS15–based micelles loaded with baicalein (HS15–BA) not only markedly increase baicalein bioavailability but also enhance its therapeutic efficacy in disease settings (Zhang et al., 2024). Formulation as an amorphous solid dispersion (ASD) can substantially increase baicalein solubility and oral bioavailability, thereby strengthening its antioxidant activity and conferring improved potential to modulate the gut microbiota (Gao et al., 2024). A complex of inclusion is created between hemisuccinate β-cyclodextrin (β-CD) and baicalein, which can improve the stability and solubility of baicalein in water, paving the way for its application in various biological contexts (Lee et al., 2019) (Figure 1) (Table 1).

**TABLE 1:** Methods for improving bioavailability.

Flavonoid	Methods for improving bioavailability	References
Baicalein	Baicalein–nicotinamide (BE–NCT) nanococrystal	Pi et al. (2019)
	Solutol HS15 (HS15–BA) micelles	Zhang et al. (2024)
	Preparation of an amorphous solid dispersion (ASD)	Gao et al. (2024)
	A baicalein inclusion complex with succinylated β-cyclodextrin (β-CD)	Lee et al. (2019)
Quercetin	A mixed hydrogel formulation	Joseph et al. (2022)
	A lecithin complex	Riva et al. (2019)
	Nanotechnology	Chowdhury et al. (2024), Ma et al. (2024)
Apigenin	An emulsion (a food-grade oil-in-water (O/W) submicron emulsion)	Abcha et al. (2019)
	A nanostructured lipid carrier (NLC)	Tapeinos et al. (2017)
	An apigenin solid dispersion	Alshehri et al. (2019)
Luteolin	A microemulsion-based delivery system	Miyashita et al. (2022)
	Sodium dodecyl sulfate (SDS)-modified luteolin nanocrystals	Liu et al. (2021)
	Preparation of a phospholipid complex	Mao et al. (2025)
	β-Cyclodextrin (β-CD)	Yang et al. (2024)
EGCG	Nanotechnology	Peng et al. (2025)
	A folate-modified nanostructured lipid carrier (NLC)	Granja et al. (2019)
	An EGCG–royal jelly protein complex (EGCG–RJ protein complex)	Han et al. (2020)
	Esterification and glycosylation	Mehmood et al. (2022)

### 2.2 Quercetin

Quercetin (3,5,7-trihydroxy-2-(3,4-dihydroxyphenyl)-4H-chromen-4-one) is a well-recognized dietary flavonoid whose robust pharmacological activities and favourable safety profile have been widely documented. Primarily utilized as a dietary antioxidant, it also possesses anti-allergic and antiviral characteristics. This compound is included in pharmaceuticals and nutraceuticals that have received approval from regulatory authorities like the US Food and Drug Administration (USFDA) and the European Food Safety Authority (EFSA) (Joseph et al., 2022). As a plant secondary metabolite, quercetin is widely distributed across different plant tissues. Onions represent one of the richest sources, and quercetin is also abundant in foods with both dietary and medicinal value, including grapes, apples, tomatoes and tea (Wang et al., 2022; Deepika and Maurya, 2022; Nguyen and Bhattacharya, 2022). Quercetin features five hydroxyl groups situated at the 3, 5, 7, 3′, and 4′positions on its flavonoid structure, with its biological activity primarily linked to the presence of its reactive phenolic hydroxyls and the conjugated system of double bonds (Nguyen and Bhattacharya, 2022; Magar and Sohng, 2020). As a safe dietary polyphenol, quercetin has been widely studied for antioxidant, anti-inflammatory and antiviral activities, as well as for neuroprotection and the prevention and management of cardiovascular disease, underscoring its considerable clinical potential (Wang et al., 2022). However, despite being an important plant-derived compound, quercetin is persistently limited by low bioavailability, and multiple formulation strategies are being pursued to improve its aqueous solubility and stability (Sun et al., 2020).

Most of quercetin’s benefits depend on its absorption in humans; however, absorption efficiency in healthy adults is relatively low (Burak et al., 2017). Quercetin has a short circulating half-life and is rapidly cleared from the bloodstream (Dabeek and Marra, 2019). Quercetin demonstrates sensitivity to pH levels and possesses hydrophobic properties, which makes it susceptible to precipitation within the gastrointestinal system, consequently diminishing its bioavailability and accessibility (Rodrigues et al., 2022). Additionally, the limited solubility of quercetin in water hinders its absorption within the small intestine. The significant metabolism that occurs in both the liver and intestinal epithelium also diminishes systemic availability, transforming quercetin into metabolites that typically exhibit lower bioactivity. Quercetin undergoes significant first-pass metabolism in the small intestine, colon, liver, and kidneys, where it is mainly transformed into glucuronide and sulfate derivatives. In addition, the gut microbiota can further shape quercetin absorption and biotransformation (Frenț et al., 2024; Li et al., 2016). In addition, environmental factors such as light and heat can compromise the stability and content of dietary quercetin, thereby altering its bioavailability. Beyond these factors, quercetin intake and exposure are influenced by its chemical structure, the food matrix, and the timing and dose of consumption (Zhu X. et al., 2024).

To address quercetin’s poor bioavailability, multiple strategies have been explored. The creation of a hybrid hydrogel featuring quercetin (FQ-35), utilizing fenugreek galactomannan as the scaffold, was illustrated via physicochemical characterization to successfully encapsulate crystalline quercetin within the matrix of the hydrogel. This leads to a naturally occurring, self-emulsifying, and reversible delivery system for the hybrid hydrogel that shows enhanced solubility. After administering a single oral dose, this system significantly enhanced the solubility, stability, and bioavailability of quercetin (Joseph et al., 2022). In addition, a quercetin phytosome delivery system formulated with food-grade lecithin substantially increased quercetin solubility and bioavailability, while also exhibiting favourable dose linearity (Riva et al., 2019). Notably, quercetin bioavailability is also shaped by the food matrix, which can markedly modulate its absorption and utilization *in vivo* through molecular interactions within complex dietary systems (Liu et al., 2025). The use of nanotechnology in the encapsulation and delivery of plant polyphenols has become increasingly prevalent. By manipulating particle size and employing surface engineering techniques, it can successfully address numerous challenges associated with traditional formulations. As a potentially transformative drug-delivery approach, nanotechnology-enabled quercetin formulations have demonstrated clear advantages within drug delivery systems (Chowdhury et al., 2024; Ma et al., 2024) (Figure 1) (Table 1).

### 2.3 Apigenin

Apigenin is among the most widely distributed flavonoids in the plant kingdom and occurs predominantly as glycosides in vegetables, fruits and plant-based beverages (Hostetler et al., 2017). Structurally, it belongs to the flavone subclass and is often present as dimeric forms; its major sources include Asteraceae plants, such as *Artemisia* species, as well as genera including *Osmanthus*, *Ginkgo* and *Paeonia* (moutan) (Salehi et al., 2019). A significant amount of research demonstrates that apigenin shows various beneficial pharmacological properties and nutritional potential, such as antioxidant activity, and has been explored as a possible treatment option for autoimmune diseases, neurodegenerative conditions, and even cancer (Ali et al., 2017; Lotha and Sivasubramanian, 2018). Furthermore, after being absorbed by the gastrointestinal system, apigenin is capable of traversing the blood–brain barrier and reaching the brain through the bloodstream, where it interacts with GABA-A receptors and influences the central nervous system (Salehi et al., 2019; Campbell et al., 2004). Additionally, research indicates that apigenin may reduce the activity of monoamine oxidase (MAO) *in vitro* within rat brain tissue. Since abnormal MAO activity is significantly linked to various neurological and psychiatric conditions, apigenin—identified as a possible MAO inhibitor—could offer therapeutic potential for depression, anxiety, andPD (Salehi et al., 2019; Sloley et al., 2000).

The effectiveness of apigenin in terms of biological activity primarily relies on its bioavailability following digestion and absorption (Wan et al., 2007). As a bioactive substance, apigenin’s efficacy is affected by factors such as its molecular structure, digestibility, the food matrix it is contained in, as well as the presence of related transporters and metabolic enzymes (Kashyap et al., 2017). Apigenin exhibits very low solubility in water (1.35 μg/mL) and has limited permeability (Zhang et al., 2012). Additionally, due to its lipophilic nature, apigenin may get inactivated in the acidic conditions of the gastrointestinal tract, leading to decreased bioavailability and restricting its use in medicinal formulations and functional food products (Adel et al., 2022). In the intestinal mucosa, apigenin is converted into high-molecular-weight glucuronide conjugates, a process regarded as one of the central determinants of its bioavailability (Wang et al., 2019). Reports also indicate that apigenin bioavailability and tissue distribution are influenced by conjugates formed through methylation, sulfation and glucuronidation (Ng et al., 2004).

To improve apigenin bioavailability, extensive work has focused on formulation strategies. Emulsification is a commonly used approach for apigenin delivery: encapsulation within food-grade oil-in-water (O/W) submicron emulsions can increase its solubility and thereby enhance bioavailability (Abcha et al., 2019). Colloidal systems for drug delivery, known as nanostructured lipid carriers (NLCs), combine the key advantages of solid lipid nanoparticles (SLNs) and oil-in-water (O/W) nanoemulsions, simultaneously mitigating the inherent limitations present in both forms. Due to their ability to traverse the blood-brain barrier (BBB), NLCs have been the focus of significant research aimed at addressing neurological disorders, including Alzheimer’s and PD (Tapeinos et al., 2017). Orally administered apigenin NLCs achieve approximately fivefold higher bioavailability than free apigenin, and no adverse effects have been observed in experimental animals following oral dosing (Dutta et al., 2018). Beyond these approaches, apigenin solid dispersions formulated with Pluronic F-127—prepared using microwave-assisted or melt methods—have also markedly improved oral bioavailability, effectively addressing apigenin’s intrinsically low bioavailability (Alshehri et al., 2019) (Figure 1) (Table 2).

**TABLE 2:** The effect of flavonoids on PD.

Compound	Dose	Model	Target	References
Dopamine				
Baicalein	300 mg/kg	c57	DA, DOPAC, HVA, 5-HT, 5-HIAA, NE, IFN-γ, TNF-α, IL-1β, IL-2, IL-4, IL-5, IL-6, IL-10, IL-12, α-synuclein, BDNF, CREB, TrkB	Zhao et al. (2021)
Quercetin	1,10,20,50,100 μM	PC12 cell	ROS,MDA,mt-Keima,mt-Rosella,TH,α-syn,PINK1	Wang et al. (2021)
​	10 mg/kg,30 mg/kg	SD rats	ROS,MDA,mt-Keima,mt-Rosella,TH,α-syn,PINK1	Wang et al. (2021)
Apigenin	10 mg/kg,20 mg/kg	SD rats	Na+/k + ATPase,Ca2+ ATPase,AChE,TH,BDNFmRNA, GDNFmRNA,TNF-a,IL-6, NF-KB,iNOS-1,D2R,a-synuclein	Anusha et al. (2017)
EGCG	25 mg/kg	C57	DA,DOPAC, Protein Carbonyls,FPN,DMT1,Hepcidin	Xu et al. (2017)
Luteolin	25,50 mg/kg	Wistar rats	DA,GSH,Nitrite,TNF-α,Bax	Chib et al. (2025)
Glutamate				
Baicalein	10 mg/kg	C57B/6 mice	GluR1,IL-1β,TNF-α	Xue et al. (2014)
Quercetin	25 mg/kg,50 mg/kg	Wistar rats	Glu,GABA,DA,NE,5-HT,MDA,Nitrite,GSH,mitochondrial complexes I,TNF-α,IL-1β,IL-6	Sharma et al. (2020)
Apigenin	25,50 mg/kg	Wistar rats	Glu,DA,GABA, Nitrite,MDA,SOD,TNF-α,IL-1β,IL-6,caspase-1,CAT,GSH	Patel and Singh (2022)
Luteolin	20 µM	SD rats	Glu,Ca2+,ERK1/2,Synapsin I	Lin et al. (2011)
EGCG	1,5,10,20,50,100 μM	SD rats	Glutamate, EAAT2,TBARS	Yu et al. (2010)
5-HT				
Baicalein	200 mg/kg	C57BL/6	5-HT,5-HIAA,DA,DOPAC,SOD,GSH-Px,MDA,TH,DAT,GFAP	Cheng et al. (2008)
Quercetin	50 mg/kg	Wistar rats	5-HT,5-HIAA,DA,DOPAC,ACh,AChE,MDA,GSH,SOD,GPx,CAT	Madiha et al. (2021)
EGCG	50 mg/kg	Wistar rats	5-HT	Wu et al. (2021)
Apigenin	3,10,30 mg kg	C57BL/6J mice	5-HT,NA,DA,5-HIAA,MAO-A/B	Li et al. (2020)
Luteolin	10,20 mg/kg	SD rats	5-HT,DA,NE,BDNF	Tongtong et al. (2024)
​	200 μM	Primary culture of hippocampal neurons	BDNF,NT-3,TrkB,p-CREB	Tongtong et al. (2024)
ACh				
Baicalein	5 mg/kg,10 mg/kg	Wistar rats	AChE,Ach	Zhou et al. (2016)
Quercetin	100 mg/kg,200 mg/kg,300 mg/kg	Wistar rats	AChE,MDA,SOD,GPx,CAT	Sriraksa et al. (2012)
Apigenin	10,20 mg/kg	SDrats	AChE,TH,α-synuclein,D2R,MDA,GSH,SOD,CAT,GPx,GR,TNF-α,IL-6,NF-κB,iNOS-1,BDNF, GDNF	Shabbir et al. (2021)
EGCG	10 mg/kg	Wistar rats	AChE,ROS,GSH,GPx,NO	Biasibetti et al. (2013)
Luteolin	50,100,200 mg/kg	SD rats	ACh,ChAT, AChE,SOD,GSH-Px,MDA,Bcl-2,Bax	Yu et al. (2015)

### 2.4 Luteolin

Luteolin, which is also known as 3′,4′,5,7-tetrahydroxyflavone, is a naturally occurring secondary metabolite that is part of the flavonoid group (Gendrisch et al., 2021). It is widely distributed in nature across numerous edible plants and traditional medicinal herbs. Common dietary sources include celery, chamomile, pomegranate and carrots, among other everyday fruits and vegetables; it is also a key bioactive constituent of herbs such as mint and *Lonicera* (honeysuckle) (Lin et al., 2008). In recent times, a wealth of experimental data has demonstrated that luteolin has a wide range of bioactivities and pharmacological effects, which encompass—without being restricted to—anti-apoptotic, antioxidant, anti-inflammatory, and neuroprotective properties. These multifaceted mechanisms establish luteolin as a strong contender for therapeutic interventions in disorders associated with the central nervous system. Luteolin has specifically been proposed as a promising therapeutic option for the prevention and management of neurodegenerative disorders, psychiatric conditions including depression and anxiety, and different pathological issues like traumatic brain injury (Jayawickreme et al., 2024; Zhu M. et al., 2024). Luteolin is a pale-yellow crystalline powder with weakly acidic properties and is soluble in alkaline solutions; however, its high lipophilicity results in very low aqueous solubility (Chen et al., 2012).

Although luteolin exhibits multiple bioactivities, its beneficial effects are substantially limited by poor aqueous solubility and low bioavailability (Xu et al., 2025). In the intestine, the lipophilic luteolin aglycone can be directly taken up by enterocytes via passive diffusion, whereas luteolin glycosides must first be hydrolysed to the aglycone. Luteolin is then extensively metabolized in intestinal and hepatic cells, yielding primarily glucuronide and sulfate conjugates. Luteolin along with its metabolites is predominantly found in the gastrointestinal system and in organs like the liver and kidneys, with biliary excretion being a primary method of elimination (Daily et al., 2021; Deng et al., 2017). Additionally, research has shown that luteolin can easily penetrate the blood-brain barrier (BBB) and access the brain, which could be beneficial for managing CNS disorders (Sawmiller et al., 2014).

To overcome luteolin’s low bioavailability, researchers have developed microemulsion-based delivery systems that enhance its aqueous dispersibility and systemic exposure, providing a new avenue for improving its efficacy (Miyashita et al., 2022). Another study reported that sodium dodecyl sulfate (SDS), an effective tight-junction modulator, can be used to functionalize luteolin nanocrystals. Compared with unmodified luteolin nanocrystals, SDS modification markedly increased luteolin bioavailability, with gains exceeding those achieved by nanocrystal engineering alone (Liu et al., 2021). Beyond these approaches, formulation of luteolin as a phospholipid complex can enhance its solubility and bioactivity and has been associated with stronger protective effects in disease models (Mao et al., 2025). β-Cyclodextrin–based metal–organic frameworks, as green carrier platforms, have also shown strong performance in enhancing luteolin solubility and bioavailability (Yang et al., 2024) (Figure 1) (Table 1).

### 2.5 EGCG

Epigallocatechin gallate (EGCG) stands out as the most thoroughly researched natural polyphenol found in green tea, playing a significant role in its pharmacological effects. When isolated, EGCG manifests as a white to pale pink powder or crystalline solid devoid of any odor. Tea, one of the most widely consumed beverages worldwide, has its origins in China, with green tea garnering particular interest due to its substantial EGCG content. A variety of research, encompassing *in vitro*, cellular, and *in vivo* investigations, illustrates that EGCG possesses numerous biological functions, including antioxidant, anticancer, antidiabetic, anti-inflammatory, antibacterial, and neuroprotective effects. Notably, EGCG can cross the blood–brain barrier and has shown significant therapeutic promise and effectiveness in several preclinical studies (Capasso et al., 2025). As the most bioactive catechin in green tea, EGCG owes much of its potency to its distinctive chemical structure, which confers a high electron-donating capacity (Ahmad et al., 2014).

EGCG is limited by pharmacokinetic liabilities, including poor aqueous solubility and membrane permeability, rapid *in vivo* metabolism, low chemical stability and extremely low oral bioavailability, which together have constrained its clinical translation in humans (Sahadevan et al., 2023). Moreover, the extent of EGCG’s low oral bioavailability varies substantially across species. EGCG is quickly taken up by the gastrointestinal system, spread throughout the body, and primarily processed in the liver and colon; it also experiences enterohepatic circulation, which allows for some reabsorption (Van Amelsvoort et al., 2001). The relatively low bioavailability of catechins is probably due to significant metabolic processes in the gastrointestinal tract and hepatic extraction following absorption. Furthermore, elements like temperature, pH levels, and the presence of metal ions may adversely affect their stability (Capasso et al., 2025; Zhu et al., 2000).

To address EGCG’s poor bioavailability, nanotechnology-based strategies—by encapsulating EGCG within nanoparticles—can markedly improve its chemical stability and pharmacokinetic profile, thereby increasing systemic exposure and therapeutic potential (Peng et al., 2025). In addition, folate-functionalized nanostructured lipid carriers (NLCs) have been proposed as safer and more efficient vehicles for EGCG, improving its pharmacokinetic performance following oral administration and ultimately enhancing its pleiotropic health benefits (Granja et al., 2019). Complexes formed between EGCG and royal jelly proteins (EGCG–RJ protein complexes) are expected not only to improve EGCG bioavailability but also to enhance its bioactivities, offering a novel strategy to optimize EGCG delivery *in vivo* (Han et al., 2020). Chemical modifications such as esterification and glycosylation also offer feasible routes to improve EGCG bioavailability (Mehmood et al., 2022) (Figure 1) (Table 1).

## 3 Toxicology

Although natural flavonoids can, under appropriate conditions, act as multi-feature, multi-target agents with potential utility across a range of diseases—thereby motivating sustained efforts to develop and harness them—doses that exceed a critical threshold may conversely pose risks to human health (Tang and Zhang, 2022). Both *in vitro* and *in vivo* studies have indicated that some flavonoids can induce mutagenic effects and may even promote the proliferation of tumour cells; given the structural diversity of flavonoids, the underlying pro-carcinogenic mechanisms remain to be elucidated (Jodynis-Liebert and Kujawska, 2020). Flavonoids may also influence thyroid toxicity; however, this effect depends on multiple contextual factors—including timing of intake and real-world exposure conditions—and can thereby interfere with thyroid function and metabolism (Baldissarelli et al., 2016; Giuliani, 2019; Habza-Kowalska et al., 2019). It has been observed by researchers that certain flavonoids derived from plants may show signs of hepatotoxicity, possess pro-oxidant properties, and have possible estrogenic activity (EA) (Galati and O'Brien, 2004). Flavonoids might exhibit estrogenic activity (EA) due to the resemblance of their chemical structures to that of the endogenous estrogen 17β-estradiol (E_2_). Isoflavones in particular are often referred to as phytoestrogens and may influence normal hormonal homeostasis in children and adolescents (Zhang and Wu, 2022). However, when exposure is kept within an appropriate dose range, these risks are likely to be manageable (Othman et al., 2025). In acute oral toxicity studies in mice, flavonoid-rich extracts produced no mortality or overt toxic signs even at doses as high as 9.0 g/kg, suggesting a favourable acute safety profile (Wu et al., 2023). In clinical translation, rigorous safety evaluation of flavonoids is essential, with particular emphasis on dose selection, definition of the therapeutic window and control of long-term toxicity risks. Rational dosing should be informed not only by pharmacokinetic and toxicological data, but also by metabolic, distribution and excretion profiles *in vivo*, to minimize the likelihood of adverse effects and toxic reactions. With continued technological advances, interdisciplinary integration is becoming central to more precise and individualized safety assessment. For example, combining high-throughput screening (HTS) datasets with computational toxicology models can help predict both biological activity and potential toxicity of flavonoids, enabling rapid identification of structure–activity relationships and safety liabilities (Firma et al., 2021).

## 4 The impact on neurotransmitters

### 4.1 Dopamine

The defining feature of PD is the depletion of neurons that produce dopamine in the brain (Ramesh and Arachchige, 2023). The progressive decline of dopaminergic neurons found in the substantia nigra pars compacta (SNpc) plays a crucial role in the development of both motor and non-motor symptoms linked to this disorder (Vázquez-Vélez and Zoghbi, 2021). The substantia nigra (SN) is a subcortical nucleus situated in the midbrain, which belongs to the basal ganglia and is crucial for the regulation of movement and reward. Traditionally, it is divided into two primary subdivisions based on its structure and functions: the substantia nigra pars compacta (SNpc), located in a more dorsal position, which is home to dopaminergic neurons, and the more ventral substantia nigra pars reticulata (SNr), which has a lower concentration of neuromelanin (NM)-containing neurons and is mainly made up of GABAergic neurons (Lee et al., 2007). Dopamine is a pivotal neurotransmitter in the brain, with dopamine receptors distributed across multiple regions. Notably, dopamine receptor 2 (D2R) is enriched in basal ganglia circuits, including the substantia nigra pars reticulata (SNr), and is also prominently expressed in the ventral tegmental area (VTA) (Burns et al., 2019). Dopamine (DA) is essential for numerous significant signaling pathways within the central nervous system (CNS) and contributes to a diverse range of physiological functions, including motor control, cognitive activities, emotional reactions, and reward systems (Wise et al., 2022). The regulation of dopaminergic signaling, including its distribution and breakdown, plays a vital role, as any impairment of dopaminergic neurons may lead to various disorders, such as PD. The coordinated activities of tyrosine hydroxylase (TH), vesicular monoamine transporter 2 (VMAT2), and the dopamine transporter (DAT) primarily regulate the synthesis, release, and presence of dopamine within the extracellular environment. Excess cytosolic dopamine—or imbalances in dopamine intermediates—can pose substantial challenges for neuronal integrity; accumulation of 3,4-dihydroxyphenylacetaldehyde (DOPAL) can drive protein modifications, including promotion of α-synuclein oligomerization (Wise et al., 2022; Meiser et al., 2013). Research indicates that cytosolic dopamine is prone to oxidation, generating reactive oxygen species (ROS) and dopamine-derived quinones (DAQs), including DA o-quinone and aminochrome, which contribute to neurotoxicity (Zhou et al., 2023). At physiological pH, dopamine quinone (DAQ) is highly unstable in the cytosol and is rapidly converted into more stable intermediates—5,6-dihydroxyindole (DHI) and 5,6-indolequinone—which can ultimately polymerize and form covalent adducts with proteins, thereby promoting the synthesis of neuromelanin (NM) (Ferrari et al., 2017; Muñoz et al., 2012). Imbalance in neuromelanin can disrupt cellular homeostasis and thereby promote neurodegenerative changes (Nagatsu et al., 2022). In PD, the primary motor features, including bradykinesia and resting tremor, primarily result from the progressive degeneration of dopaminergic neurons located in the substantia nigra pars compacta (SNpc), with approximately 50%–70% having been lost by the point at which clinical symptoms begin to appear (Damier et al., 1999).

In a rotenone-induced mouse model of PD with depression-like behaviours, continuous baicalein treatment for 4 weeks markedly alleviated abnormal behavioural phenotypes. Neurochemical evaluations revealed that exposure to rotenone resulted in a notable reduction of dopamine (DA), serotonin (5-hydroxytryptamine; 5-HT), and noradrenaline (NE) levels in various brain areas, such as the cortex, striatum, and brainstem. Following the administration of baicalein, the concentrations of these monoamine neurotransmitters, as well as their primary metabolites, 3,4-dihydroxyphenylacetic acid (DOPAC) and 5-hydroxyindoleacetic acid (5-HIAA), returned to baseline levels. Additionally, baicalein mitigated neuroinflammatory reactions, inhibited the abnormal aggregation of α-synuclein, and stimulated the BDNF–TrkB–CREB signaling pathway. Collectively, these findings suggest that baicalein engages coordinated multi-target and multi-pathway actions to counteract rotenone-induced neurotransmitter disequilibrium and associated pathology, thereby improving neurobehavioural deficits in experimental PD (117). Quercetin alone does not markedly affect tyrosine hydroxylase (TH) expression. However, in a 6-hydroxydopamine (6-OHDA)-induced dopaminergic injury model, TH levels are substantially reduced, and treatment with 20 μM quercetin partially reverses this decrease while concomitantly lowering α-synuclein expression. Notably, as the rate-limiting enzyme in dopamine biosynthesis, TH expression provides a direct readout of dopaminergic neuronal functional integrity. Rather than directly elevating dopamine levels, quercetin appears to protect dopaminergic neurons from 6-OHDA toxicity by activating the PINK1–Parkin pathway and strengthening mitochondrial quality control, thereby preserving TH expression and maintaining the capacity for dopamine synthesis (Wang et al., 2021). In a rotenone (ROT)-induced animal model of PD, tyrosine hydroxylase (TH) immunostaining is markedly reduced, indicating dopaminergic neuronal injury. Following treatment with apigenin (AGN), the decline in TH protein levels is substantially attenuated, suggesting that AGN may modulate the dopaminergic system—potentially by supporting dopamine biosynthesis—while concurrently ameliorating α-synuclein pathology. In addition, AGN upregulates dopamine D2 receptor (D2R) expression and influences cholinergic signalling, including modulation of acetylcholinesterase (AChE). Beyond its effects on neurotransmission, AGN also reduces inflammatory cytokines (Anusha et al., 2017). Dopamine performs its roles within the central nervous system via dopamine receptors, classified into two subfamilies: D1-like receptors (including D1 and D5) and D2-like receptors (comprising D2, D3, and D4). Notably, D2R stands out as the most prevalent and abundantly expressed receptor within the D2-like category, with its levels significantly surpassing those of D3 and D4 (Missale et al., 1998). Previous studies have shown that rotenone (ROT) induces neurotoxicity, leading to downregulation of D2R expression, which is closely linked to neurodegenerative progression in PD (Siddiqui et al., 2010). Notably, iron overload is considered one of the key drivers of dopaminergic neurodegeneration (Rathnasamy et al., 2013). EGCG (epigallocatechin gallate) can upregulate the iron-export protein ferroportin (FPN), thereby promoting neuronal iron efflux and alleviating iron-driven oxidative stress. EGCG has been shown to significantly reduce dopaminergic neurotoxicity induced by MPTP, possibly due to its combined actions that are anti-inflammatory and antioxidant. In one study, EGCG treatment increased striatal dopamine concentrations by ∼40% relative to the MPTP model group, indicating a partial reversal of dopamine depletion. In the present work, FPN expression in the substantia nigra was ∼44% higher in EGCG-treated mice than in the MPTP group, suggesting that enhanced control of iron homeostasis may represent an important mechanism by which EGCG protects the dopaminergic system. Thus, rather than directly elevating dopamine, EGCG may act indirectly by suppressing pathological processes that drive dopaminergic neuronal injury (Xu et al., 2017). In a rotenone-induced Wistar rat model, luteolin significantly increased brain dopamine levels while concurrently alleviating oxidative stress, suppressing neuroinflammation and reducing apoptosis, consistent with a multi-target therapeutic profile (Chib et al., 2025) (Table 2).

### 4.2 Glutamate

Glutamate serves as a fairly prevalent excitatory neurotransmitter crucial for the proper operation of the nervous system. Recent studies indicate that Parkison’s patients exhibit higher serum levels of glutamate compared to healthy individuals (Zhou and Danbolt, 1996). Sustained glutamate excitotoxicity—driven by NMDA receptor activation and excessive intracellular Ca^2+^ influx—represents a high-risk mechanism underlying glutamate-mediated neuronal injury (Yang et al., 2014). Glutamate modulates synaptic plasticity, shapes connectivity between neurons, and is essential for normal neurotransmission (Alix and Domingues, 2011). Glutamate is extensively found within the brain and is vital for cognitive processes, including learning and memory (McEntee and Crook, 1993). In the nervous system, neurotransmission is initiated by glutamate, which attaches to particular receptors and leads to alterations in the membrane potential of neurons (Traynelis et al., 2010). Growing evidence suggests that abnormal levels of glutamate, faulty receptor functioning, and irregular transporter activity are closely connected to the disease mechanisms of PD, wherein motor dysfunction is strongly related to increased glutamate in the basal ganglia (Nandhu et al., 2011). Therapeutic targeting of glutamate receptors can not only alleviate the motor symptoms of PD but also enhance the efficacy of dopaminergic agents and confer neuroprotection (Zhang et al., 2019). In PD, the expression of glutamate transporters is markedly reduced, which exacerbates glutamate accumulation within the synaptic cleft, thereby intensifying excitotoxic injury and perpetuating disease progression (Zhang et al., 2016). During the pathophysiological course of PD, glutamate receptors within the basal ganglia and other discrete regions also undergo regulatory alterations. Such changes are likewise evident during LID; for example, following levodopa treatment, MPTP-lesioned monkeys exhibit increased NMDA receptor–specific binding alongside reduced mGlu2/3 receptor levels (Jourdain et al., 2015). Moreover, glutamate contributes to neurodegenerative processes through excitotoxic mechanisms; when cellular energy metabolism is compromised, glutamate can act as a neurotoxin, promoting the death of dopaminergic neurons (Blandini et al., 1996). The enhancement of glutamatergic transmission significantly elevates the excitability of neurons in the striatum and serves as a primary factor in neuronal death caused by excitotoxicity across various neurological conditions (Liu and Zukin, 2007). Restoration of striatal glutamatergic tone is strongly associated with improved performance of spontaneous movement in rodent models of PD (Bage et al., 2012).

Baicalein markedly attenuates aberrant central cytokine production and striatal hyperglutamatergic transmission in MPTP-induced mouse models. Mechanistically, it decreases the release of presynaptic glutamate and facilitates the insertion of the postsynaptic glutamate receptor subunit GluR1, thus effectively inhibiting the increase in basal striatal glutamatergic activity induced by MPTP. Furthermore, baicalein notably reduces the levels of pro-inflammatory cytokines in both the substantia nigra and striatum, implying that it could indirectly mitigate the inflammation-related enhancement of glutamatergic signaling by lessening neuroinflammation (Xue et al., 2014). A mechanism that is broadly similar has been observed for quercetin as well. In a rat model of PD, where rotenone coupled with iron supplementation was used to induce the condition, quercetin significantly lowered the abnormally high levels of striatal glutamate while normalizing γ-aminobutyric acid (GABA) levels, thus rectifying the imbalance between excitatory and inhibitory amino acid neurotransmission. The neuroprotective properties of quercetin appear to stem from multiple factors, including the reduction of mitochondrial dysfunction and oxidative stress, the inhibition of neuroinflammation driven by TNF-α, IL-1β, and IL-6, and the safeguarding of nigrostriatal dopaminergic neurons, which ultimately enhances dopaminergic neurotransmission. Overall, these results provide experimental evidence for quercetin’s role in sustaining neurotransmitter equilibrium (Sharma et al., 2020). In an LPS-induced animal model of PD, apigenin dose-dependently reduced alterations in striatal glutamate levels in rats, thereby counteracting excitotoxicity. Under pathological conditions, LPS activates NMDA receptors, increasing intracellular Ca^2+^ release; this Ca^2+^ efflux further engages NMDA signalling and promotes glutamate influx, culminating in glutamate-mediated excitotoxic damage. Notably, apigenin also increased striatal dopamine (DA) and γ-aminobutyric acid (GABA) levels, mitigating injury driven by excessive glutamate (Patel and Singh, 2022). Studies have shown that luteolin mitigates neurotoxin-induced neurotoxicity. It regulates the release of glutamate from isolated and purified nerve terminals (synaptosomes) obtained from the rat cerebral cortex, and the inhibition of glutamate release induced by 4-aminopyridine (4-AP) seems to be mainly facilitated by the suppression of voltage-gated calcium channels Cav2.2/Cav2.1,thereby lowering intracellular Ca^2+^ by reducing Ca^2+^ influx. Luteolin also engages the MAPK/ERK signalling pathway, ultimately shaping glutamate release. Interestingly, a variety of kinases, such as mitogen-activated protein kinases (MAPKs), protein kinase C (PKC), and protein kinase A (PKA), modulate glutamate secretion at the presynaptic level (Lin et al., 2011). Ultimately, epigallocatechin gallate (EGCG) successfully prevents glutamate excitotoxicity that is triggered by the glutamate transporter inhibitor THA. While THA administration significantly raises glutamate concentrations in the culture medium, the simultaneous application of EGCG counteracts this effect (Yu et al., 2010) (Table 2).

### 4.3 5-HT

Analyses conducted on the brains of individuals who had PD post-mortem revealed lower concentrations of striatal 5-HT and its metabolite, 5-hydroxyindoleacetic acid (5-HIAA) (Kish et al., 2008). Serotonin, also known as 5-hydroxytryptamine (5-HT), is a monoamine neurotransmitter that serves various biological roles (Li, 2023). This neurotransmitter is crucial in the advancement of neurodegeneration and related diseases (Correia et al., 2023). An imbalance in the brain’s 5-HT system has been linked to cognitive decline and the emergence of depressive symptoms in individuals suffering from PD (Mendonça et al., 2020). Furthermore, in various neurodegenerative diseases, the serotonergic system has shown significant dysregulation (Correia et al., 2021). The dysfunction of the serotonergic system has been linked to non-motor symptoms experienced in PD, including sleep disturbances, cognitive deterioration, and symptoms of anxiety and depression. Additionally, abnormalities in serotonergic neurons can affect dopaminergic neurons in the substantia nigra pars compacta (SNpc), playing a role in the development of motor symptoms.Because serotonergic neurons—and other neuronal populations involved in autonomic regulation—also undergo degeneration, non-motor symptoms often precede motor features (Khowdiary et al., 2025). Recent studies have demonstrated that 5-hydroxytryptamine influences neuronal function in the substantia nigra pars compacta (SNpc) by activating serotonin receptors, resulting in various regulatory effects (Miguelez et al., 2014). In animal studies, PD model rats exhibit reduced plasma 5-hydroxytryptamine concentrations and decreased expression of the serotonin reuptake transporter (SERT) (Rylander et al., 2010). It is important to highlight that various subtypes of 5-HT receptors—such as 5-HT1A, 5-HT2, 5-HT3, and 5-HT6—play a role not only in managing extrapyramidal motor disturbances associated with PD but also have significant impacts on cognitive abilities (Ballanger et al., 2012; Ohno et al., 2015). 5-Hydroxytryptamine influences the management of striatal L-DOPA; for instance, the antidepressant citalopram reduces levodopa-induced dyskinesia in mouse models of PD through the downregulation of SERT activity (Chen et al., 2024). Long-term treatment with serotonergic agonists lasting more than 6 months reduces levels of β-amyloid (Aβ1–42) in the cerebrospinal fluid (CSF) of patients with PD who are facing cognitive challenges, indicating a possible neuroprotective function of serotonin in lessening the cognitive decline linked to PD (Wilson et al., 2018).

Baicalein exhibits notable neuroprotective effects and is considered a potential candidate for tackling PD. Studies indicate that baicalein is capable of significantly diminishing behavioral abnormalities in mice that have received treatment with 1-methyl-4-phenyl-1,2,3,6-tetrahydropyridine (MPTP).The protective effects appear to stem from various mechanisms: it enhances levels of striatal dopamine (DA) and 5-hydroxytryptamine (5-HT), boosts the population of dopaminergic neurons, and also mitigates oxidative stress responses and astrocyte activation. Overall, these results imply that baicalein offers a neuroprotective profile targeting multiple pathways in models of PD (Cheng et al., 2008). Monoamine oxidase (MAO) plays a crucial role in the metabolism of catecholamines by facilitating the oxidative deamination of biogenic amines, including dopamine (DA), 5-hydroxytryptamine (5-HT), and noradrenaline (Zatta et al., 1999). By assessing changes in the 5-HIAA-to-5-HT ratio following baicalein intervention, the study found a reduction in this ratio, suggesting that baicalein may partially inhibit monoamine oxidase (MAO) activity and thereby reduce 5-hydroxytryptamine (5-HT) degradation, helping to maintain its homeostatic levels in the brain (Cheng et al., 2008). A similar phenomenon has been observed in rotenone-induced animal models of PD following quercetin intervention. Rotenone treatment markedly reduced levels of striatal and hippocampal 5-HT, its metabolite 5-HIAA, dopamine (DA) and 3,4-dihydroxyphenylacetic acid (DOPAC), whereas quercetin administration effectively reversed these decreases. The study further noted that quercetin not only significantly elevated 5-HT levels and ameliorated depression-like behaviours, but may do so, at least in part, by inhibiting monoamine oxidase A (MAO-A) activity. In addition, the potent antioxidant capacity of quercetin may indirectly facilitate restoration of monoaminergic neurotransmitter homeostasis by alleviating oxidative stress (Madiha et al., 2021). Studies indicate that apigenin exerts marked neuroprotective and anti-inflammatory effects in MPTP-induced animal models of PD, suggesting its therapeutic potential in PD (Yarim et al., 2025). An investigation into the impact of apigenin on the neurotransmitter 5-HT revealed that apigenin increased 5-HT levels in a dose-dependent manner while simultaneously lowering the 5-HIAA/5-HT ratio. Additionally, there was an observed rise in MAO-A activity, and apigenin was shown to work synergistically with the 5-HT precursor 5-hydroxytryptophan (5-HTP) (Wu et al., 2021). Moreover, mechanistic studies have confirmed that apigenin can significantly inhibit monoamine oxidase A (MAO-A) activity, thereby alleviating depression-like behaviours (Olayinka et al., 2023). Monoamine oxidase A (MAO-A) plays a crucial role in the deamination process of monoaminergic neurotransmitters and is found in both the brain and peripheral tissues. Additionally, it serves as an important biochemical junction associated with depression (Grunewald et al., 2012; Thorpe et al., 1987). Available evidence suggests that EGCG may confer neuroprotection in PD by modulating ferroptosis (Xia et al., 2024). Moreover, EGCG appears to exert antidepressant effects by modulating 5-HT. Analyses of hippocampal tissue showed that EGCG intervention reversed the reduction in 5-HT, indicating a central effect; by contrast, 5-HT levels in the colon did not show the same pattern. These observations suggest that EGCG-mediated regulation of 5-HT homeostasis may be linked to brain–gut interactions (Li et al., 2020) Concerning the common non-motor symptoms linked to PD, such as depression, luteolin has shown promise as a potential therapeutic option. Recent studies indicate that luteolin not only boosts the levels of serotonin (5-HT) and dopamine (DA) in synapses but also affects the expression of brain-derived neurotrophic factor (BDNF).This implies that its effects may reach beyond mere neurotransmitter modulation, possibly affecting neuroplasticity and neurodevelopment (Tongtong et al., 2024) (Table 2).

### 4.4 ACh

PD encompasses more than merely the loss of dopaminergic neurons; at the time of diagnosis, alongside the degeneration of these neurons within the substantia nigra pars compacta (SNc), there is also a decline in neurons that synthesize choline acetyltransferase (ChAT) (Nakano and Hirano, 1984; Braak et al., 2004). In PD, alterations in the cholinergic system can occur in specific regions of the brain, influencing both motor and non-motor aspects, and these clinical characteristics frequently do not respond well to dopaminergic treatment (Bohnen et al., 2022). Neuropathological examinations after death and *in vivo* imaging research have shown that changes in cholinergic function in PD correlate with dementia, episodes of falling, and issues with gait (Bohnen et al., 2018a). The decline of the cholinergic system plays a significant role in the pathophysiology of cognitive deficits associated with PD (Bohnen et al., 2018b). Cholinergic deficits selectively compromise memory, attention and visuospatial abilities (Bohnen et al., 2006; van der Zee et al., 2021). Studies indicate a notable relationship between the extent of cholinergic deficits and the level of cognitive impairment seen in PD (Hilker et al., 2005; Bohnen et al., 2015). However, acetylcholine does not invariably decline; rather, it may change over the disease course. In the early stages, acetylcholine activity in certain regions may increase compensatorily to preserve brain function, whereas with disease progression and failure of these compensatory mechanisms, acetylcholine levels subsequently decline markedly (Bohnen et al., 2022).

Available evidence indicates that baicalein dose-dependently modulates acetylcholinesterase (AChE) and acetylcholine (ACh) levels in brain tissue, thereby contributing to its neuroprotective effects. Overall, baicalein reduces AChE levels and consequently increases brain ACh content; this cholinergic modulation is considered one of the key mechanisms by which baicalein improves cognition and memory in rats (Zhou et al., 2016). Inhibition of AChE activity reduces the rate of acetylcholine degradation, thereby helping to maintain ACh levels (Colović et al., 2013). A similar phenomenon has been observed in a 6-hydroxydopamine-induced rat model, in which quercetin increased synaptic-terminal acetylcholine by significantly reducing hippocampal AChE activity; however, this inhibitory effect was evident primarily at the high dose (300 mg/kg) and was not significant at the intermediate or low doses (200 or 100 mg/kg) (Sriraksa et al., 2012). EGCG likewise influences ACh through modulation of AChE activity: EGCG treatment reduces hippocampal AChE activity in rats, restoring it towards normal levels, thereby decreasing ACh breakdown and indirectly increasing ACh levels. Notably, AChE activity may be regulated by MAPK signalling (Biasibetti et al., 2013). In functional assays utilizing cells, apigenin does not directly stimulate the α7 nicotinic acetylcholine receptor (α7 nAChR); instead, it functions as a positive allosteric modulator that enhances the Ca^2+^ response mediated by the α7 receptor when acetylcholine (ACh) is present. This indicates potential neuromodulatory benefits through improved efficiency of cholinergic signaling (Shabbir et al., 2021). In animal models treated with luteolin, luteolin reversed the increase in hippocampal acetylcholinesterase (AChE) activity while concomitantly elevating acetylcholine (ACh) levels (Yu et al., 2015) (Table 2).

## 5 Discussion

Currently, levodopa (L-DOPA) is considered the ‘gold standard’ treatment for PD; while it is effective in the short term, its long-term negative effects limit its overall utility. This highlights the pressing necessity to investigate alternative or supplementary approaches. In this Review, we focus on the pharmacological impacts of flavonoid compounds such as baicalein, quercetin, apigenin, luteolin, and epigallocatechin gallate (EGCG) within the framework of PD. These flavonoids have been confirmed through various mechanisms, which include, but are not restricted to, their antioxidant and anti-inflammatory properties. Notably, they possess the ability to influence neurotransmission positively, showing advantageous impacts on dopamine, 5-HT, glutamate, and acetylcholine. As naturally occurring substances frequently found in various foods, flavonoids have become key ingredients in functional foods, medicinal applications, and pharmaceutical innovations.

Baicalein markedly reverses rotenone-induced reductions in dopamine (DA) levels in the cortex, striatum and brainstem, suppresses α-synuclein aggregation, and activates the BDNF–TrkB–CREB signalling axis, thereby protecting nigral dopaminergic neurons. Quercetin enhances mitochondrial quality control via activation of the PINK1–Parkin pathway, preserving tyrosine hydroxylase (TH) expression and, indirectly, sustaining DA biosynthetic capacity. Apigenin improves dopaminergic pathway function by upregulating dopamine D2 receptor (D2R) expression and modulating acetylcholinesterase (AChE) activity. Epigallocatechin gallate (EGCG) alleviates iron overload–driven oxidative stress through upregulation of the iron exporter ferroportin, indirectly safeguarding dopaminergic neurons and increasing striatal DA concentrations by ∼40%.Baicalein, quercetin and apigenin also inhibit monoamine oxidase A (MAO-A), reduce the 5-hydroxyindoleacetic acid (5-HIAA)/5-HT ratio and markedly elevate brain 5-HT levels, thereby mitigating PD-associated symptoms. EGCG, in turn, bi-directionally regulates 5-HT concentrations in the central nervous system and colon via the brain–gut axis, indirectly improving neurological function.With respect to glutamatergic signalling, baicalein reduces presynaptic glutamate release and promotes postsynaptic GluR1 insertion, dampening glutamatergic hyperactivity. Quercetin lowers striatal glutamate levels and restores GABA balance in iron-overload models, whereas apigenin and luteolin counter excitotoxicity by suppressing NMDA receptor activation and inhibiting calcium channel–dependent glutamate release, respectively. EGCG dose-dependently attenuates glutamate-induced neuronal apoptosis, potentially involving hippocalcin regulation.Finally, baicalein, quercetin, EGCG and luteolin increase acetylcholine (ACh) levels by inhibiting AChE activity. Apigenin, acting as a positive allosteric modulator of α7 nicotinic ACh receptors, further enhances the efficiency of cholinergic signalling and improves cognitive function.

The therapeutic efficacy of these five flavonoid compounds is constrained by poor aqueous solubility, extensive intestinal first-pass metabolism and low oral absorption. Encouragingly, strategies such as nanotechnology-based formulations and phospholipid complexes can improve their bioavailability. Although flavonoids are neuroprotective within rational dose ranges, suprathreshold intake may elicit hepatotoxicity, pro-oxidant effects and oestrogenic activity (particularly for isoflavones), issues that warrant further investigation. Future prospects for clinical translation should therefore focus on optimizing next-generation delivery systems, refining dose design with greater precision, and advancing clinical validation by translating findings from animal models into randomized controlled trials in humans to more clearly define efficacy and safety.

## 6 Summarize

In this Review, we aimed to clarify whether flavonoids could provide therapeutic value in PD from a multi-neurotransmitter perspective rather than through a dopamine-centred framework alone. By integrating available evidence on five representative flavonoids—baicalein, quercetin, apigenin, luteolin and epigallocatechin gallate (EGCG)—we found that these compounds exert convergent neuroprotective effects across several neurotransmitter systems, particularly dopamine, 5-hydroxytryptamine (5-HT), glutamate and acetylcholine. Collectively, the reviewed studies indicate that flavonoids may preserve dopaminergic function, reduce glutamate-driven excitotoxicity, improve serotonergic and cholinergic signalling, and ameliorate both motor and non-motor manifestations of PD. Mechanistically, these effects appear to involve not only anti-oxidative and anti-inflammatory actions, but also regulation of α-synuclein pathology, mitochondrial quality control, monoamine metabolism, receptor-related signalling and iron metabolism. This integrated view represents a key conceptual contribution of the present Review, highlighting that flavonoids may offer a broader non-dopaminergic strategy for PD management than single-transmitter intervention alone. From a translational perspective, however, their clinical potential remains constrained by poor aqueous solubility, extensive first-pass metabolism, low oral bioavailability and the need for more rigorous safety evaluation. Future research should therefore prioritize optimized delivery systems, pharmacokinetic refinement, dose–response characterization, and well-designed clinical investigations to determine whether these promising preclinical effects can be translated into effective and safe therapies for patients with PD.
